# Sequence-structure relationships, expression profiles, and disease-associated mutations in the paralogs of phosphoglucomutase 1

**DOI:** 10.1371/journal.pone.0183563

**Published:** 2017-08-24

**Authors:** Andrew G. Muenks, Kyle M. Stiers, Lesa J. Beamer

**Affiliations:** Biochemistry Department, University of Missouri, Columbia, Missouri, United States of America; University of Michigan, UNITED STATES

## Abstract

The key metabolic enzyme phosphoglucomutase 1 (PGM1) controls glucose homeostasis in most human cells. Four proteins related to PGM1, known as PGM2, PGM2L1, PGM3 and PGM5, and referred to herein as paralogs, are encoded in the human genome. Although all members of the same enzyme superfamily, these proteins have distinct substrate preferences and different functional roles. The recent association of PGM1 and PGM3 with inherited enzyme deficiencies prompts us to revisit sequence-structure and other relationships among the PGM1 paralogs, which are understudied despite their importance in human biology. Using currently available sequence, structure, and expression data, we investigated evolutionary relationships, tissue-specific expression profiles, and the amino acid preferences of key active site motifs. Phylogenetic analyses indicate both ancient and more recent divergence between the different enzyme sub-groups comprising the human paralogs. Tissue-specific protein and RNA expression profiles show widely varying patterns for each paralog, providing insight into function and disease pathology. Multiple sequence alignments confirm high conservation of key active site regions, but also reveal differences related to substrate specificity. In addition, we find that sequence variants of PGM2, PGM2L1, and PGM5 verified in the human population affect residues associated with disease-related mutants in PGM1 or PGM3. This suggests that inherited diseases related to dysfunction of these paralogs will likely occur in humans.

## Introduction

The human genome contains five proteins in the **α**-D-phosphohexomutase superfamily. They are epitomized by phosphoglucomutase 1 (PGM1), a critical enzyme in metabolism that regulates glucose homeostasis through the interconversion of glucose 1-phosphate and glucose 6-phosphate [1]. Four other proteins, known as PGM2, PGM2L1, PGM3, and PGM5, are sequence related to PGM1, but differ in their substrate preferences or mechanism, and have distinct biological roles. PGM2 has phosphopentomutase activity, while PGM2L1 (for PGM2-like 1) is specialized for glucose 1,6-bisphosphate (G16P) synthase activity [2]. PGM3 is an N-acetylglucosamine phosphomutase [3] and participates in hexosamine biosynthesis. PGM5 is reported to lack enzyme activity [4], but has established structural roles in myofibril assembly and maintenance [5], and is a binding partner for dystrophin [6].

All of these proteins belong to the **α**-D-phosphohexomutase superfamily, which is ubiquitous in all kingdoms of life [7,8]. Members of the superfamily typically catalyze an intramolecular phosphoryl transfer, often from the 1- to the 6-position of a hexose. Major sub-groups within the enzyme superfamily differ in their preferences for the sugar moiety of their substrates, which include glucose, mannose, glucosamine and N-acetyl glucosamine. In eukaryotes, several other minor sub-groups of the superfamily have been described, including proteins with phosphopentomutase and G16P synthase activities [2]. Sequence analyses clearly show that all the human PGM1 paralogs share the highly conserved active site residues characteristic of the superfamily [2–4]. These include the catalytic phosphoserine involved in phosphoryl transfer and a binding site for a metal ion required for maximal activity, as well as residues that participate in sugar and phosphate interactions [1,7].

Although previous studies provided insights into the PGM1 paralogs, overall these proteins are not well characterized, especially compared to PGM1. Only a few biochemical studies using recombinant, purified versions of the proteins have been conducted [2,3,5]. Moreover, previous evolutionary and expression analyses, which are key to an appreciation of their functional roles, preceded the dramatic increase of genomic and expression data. Finally, a better understanding of the human paralogs of PGM1 is timely, given the recent recognition of enzyme deficiencies of PGM1 and PGM3 as the cause of inherited diseases in humans [9–14]. PGM1 deficiency has features of both a glycogen storage disease and a congenital disorder of glycosylation, of types I and II. PGM3 deficiency is also a congenital disorder of glycosylation, with symptoms that include immunodeficiency and neurocognitive impairment. In both diseases, patient phenotypes are variable and complex, affecting multiple tissues and organ systems.

Here we further explore sequence, structure, and tissue-specific expression relationships between PGM1 and its four paralogs. This information is relevant to understanding their biochemical and biological roles. We also identify variants in each of these five proteins known to exist in the human population that are likely to cause enzyme dysfunction. This suggests that, in addition to PGM1 and PGM3, inherited disorders may be linked to missense variants of the other paralogs.

## Results

### Overview of functional relationships

Genetic loci for PGM1 and two related “isozymes” (PGM2 and PGM3) were first were identified in the 1960’s. Early kinetic studies [15,16] showed that PGM1 was a highly effective phosphoglucomutase, while PGM2 was most effective catalyzing a phosphoribomutase reaction. Several decades later, human PGM3 was found to be the same as AGM1, an N-acetylglucosamine-phosphate mutase [3,17]. The molecular activities of recombinantly-expressed PGM2 and PGM2L1 were characterized in 2007, defining them as a phosphoribomutase and G16P synthase, respectively [2]. In 1994, a protein called aciculin was first described [18], which was later identified as a member of phosphoglucomutase superfamily, and is now called PGM5. Although the evolutionary relationships of these proteins are complex (see following sections), we use the term paralog herein (rather than homolog or ortholog) to emphasize the presence of five functionally distinct proteins within the genome.

An overview of the PGM1 paralogs, summarizing key biochemical data, is shown on Table 1. As evident from their EC numbers, PGM1, PGM2, and PGM3 are isomerases, utilizing a phosphoserine residue of the protein in the reversible conversion of mono-phosphosugars (e.g., 1- to -6-phospho) via a bisphosphorylated sugar intermediate (e.g, glucose 1,6-bisphosphate). While mechanistically similar, these enzymes differ in their preference for the sugar moiety of their substrates, as noted above. PGM2L1 differs from the other paralogs by catalyzing a distinct reaction, utilizing glucose 1-phosphate and 1,3-bisphospho-glycerate as substrates to produce G16P and 3-phosphoglycerate [2].

**Table 1:** Summary of the human PGM1 homologs.

Paralog (EC No.)	Name	Lineage*	UniProtKB	PDB entry (organism)	No. resi	pI
**PGM1** (5.4.2.2)	phosphoglucomutase	animals, plants, fungi	P36871	5EPC (human)	562	6.3
**PGM2** (5.4.2.7)	phosphopentomutase	animals, fungi	Q96G03	-	612	6.3
**PGM2L1** (2.7.1.106)	G16P synthase	vertebrates	Q6PCE3	-	622	6.8
**PGM3** (5.4.2.3)	phosphoacetyl-glucosamine mutase	animals, plants, fungi	O95394	2DKA, 2DKC, 2DKD (*C*. *albicans*), 4BJU (*A*. *fumigatus*)	542	5.8
**PGM5**	aciculin	vertebrates	Q15124	-	567	6.8

*Only eukaryotic lineages included.

Sequence ID, no. residues, and pI correspond to the major transcript of each protein. EC: Enzyme Commission; PDB: Protein Data Bank.

Also on Table 1 are the overall distributions of the paralogs in eukaryotic organisms: PGM1 and PGM3 are the most broadly distributed, while PGM2L1 and PGM5 are the most restricted, found only in vertebrates [2,19]. PGM2 is also widely found in eukaryotes, but appears to be missing in plants [2]. Sequence lengths and calculated isoelectric points are listed for the major transcript of each protein. Except for PGM2L1, all of the paralogs have alternative transcripts (e.g., see annotations in the ExAC database: exac.broadinstitute.org), most of which are truncations and likely non-functional. PGM1, however, is known to have a functional, alternatively spliced form that is expressed in fast muscle [20]. All of the paralogs appear to be cytosolic, and several are involved in protein-protein interactions important in a range of cellular functions [4–6,18,21,22].

### Sequence-structure relationships

**Fig 1:**
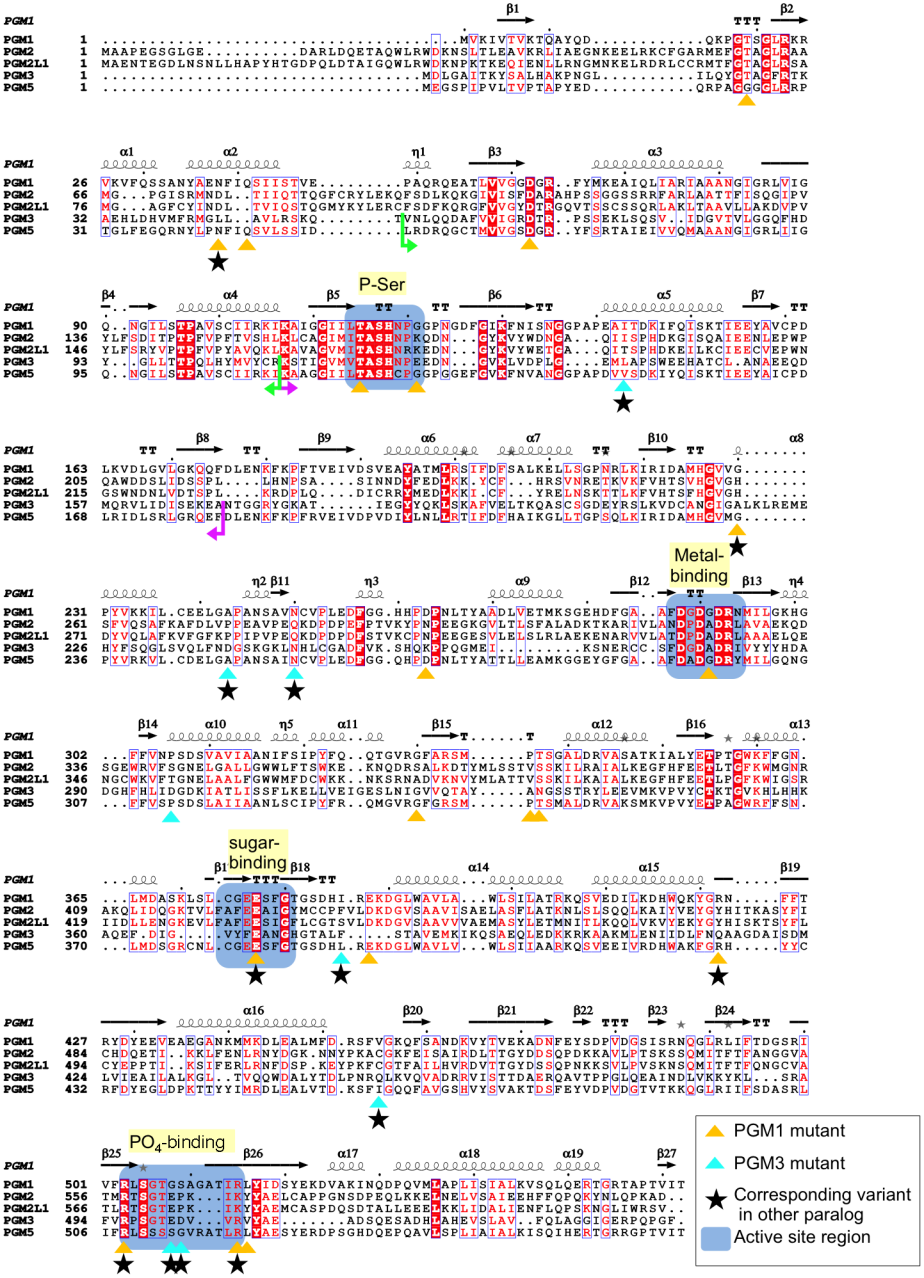
A multiple sequence alignment of human PGM1 and its paralogs. Strictly conserved residues are in white font on a red background; highly conserved residues are in red font. Secondary structure elements from the human PGM1 crystal structure (PDB-ID 5EPC) are above the alignment: **α**-helices are shown as coils, **β**-strands as arrows, and turns by TT. Residues with known disease-associated missense variants are highlighted with triangles (gold for PGM1, cyan for PGM3). Residues with variants from the ExAC database corresponding to disease-related mutants in PGM1 or PGM3 are indicated with black star. For other symbols, see key on figure. The amino acid sequence of PGM3 has been altered to account for its circular permutation relative to the other proteins; borders of translocated segments (~60 aa) are indicated by green/pink arrows (see also S1 Fig).

Sequence relationships between the PGM1 paralogs are readily apparent, due to the strongly conserved active site motifs found in the entire enzyme superfamily. These are highlighted on Fig 1, and include four key regions: i) the phosphoserine residue required for phosphoryl transfer; ii) the metal-binding loop; iii) the sugar-binding loop; and iv) the phosphate (PO_4_)-binding site that interacts with the phosphate group of the substrate. Despite considerable overall sequence diversity (see following sections), the conservation of these functional regions clearly establishes these proteins as members of the **α**-D-phosphohexomutase superfamily.

**Fig 2:**
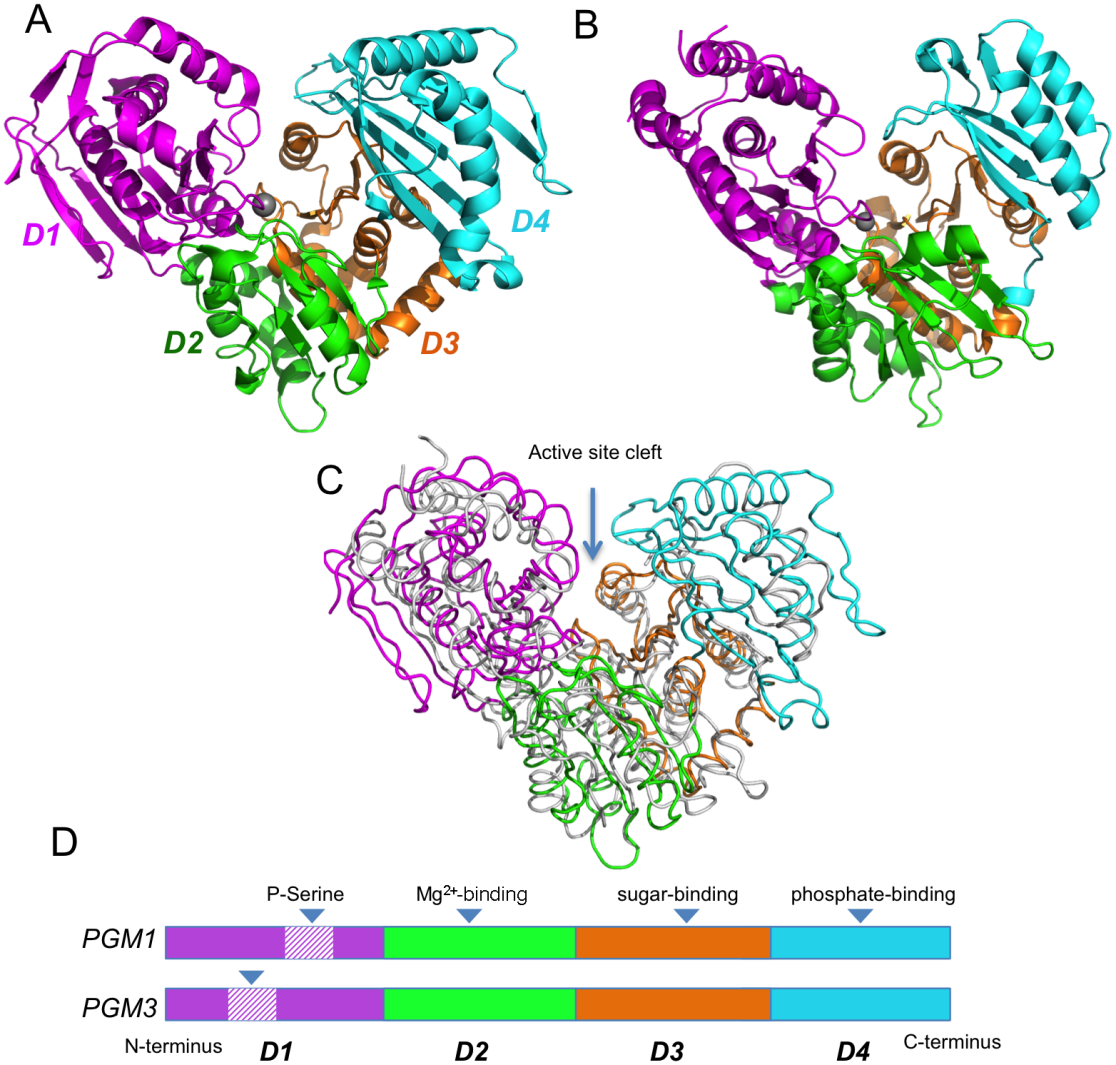
Crystal structures of human PGM1 and a PGM3 homolog. **(A)** Human PGM1 (PDB ID 5EPC) colored by structural domain: domain 1 (magenta, residues 1–191); domain 2 (green, residues 192–304); domain 3 (orange, residues 305–421), and domain 4 (cyan, residues 422–562). Bound metal ion in the active site is shown as gray sphere. **(B)** A homolog of PGM3 (*N*-acetylphosphoglucosamine mutase from *C*. *albicans*; PDB ID 2DKD) colored as in (A). The domains are: domain 1 (residues 1–191); domain 2 (residues 192–311); domain 3 (residues 312–456); and domain 4 (residues 457–544). (**C)** A structural superposition of PGM1 (colored by domain) and the PGM3 homolog (white). **(D)** A schematic of the domain arrangement of PGM1 and PGM3, indicating the residues transposed by the circular permutation in domain 1 of PGM3. Key active site regions are indicated.

Although only one of the human enzymes, PGM1 (PDB ID 5EPC), has been structurally characterized, the three-dimensional (3D) structure of two PGM3 orthologs from *Candida albicans* (PDB IDs 2DKA, 2DKC, 2DKD) and *Aspergillus fumigatus* (PDB ID 4BJU) are known. Comparison of 5EPC and 2DKD (Fig 2) shows that the sequence relationships between these proteins extend to 3D similarities, including the four-domain structural architecture, with residues in each domain contributing to key regions of the active site. The similarity in structure is particularly evident in the central, active site cleft, as would be expected from the high sequence conservation and functional importance of these regions.

Relative to the other human paralogs, PGM3 and its orthologs have a significant alteration in their primary amino acid sequence, due to a circular permutation within domain 1 [23]. This affects an approximately 60-residue segment of the protein, which includes the functionally critical phosphoserine loop. However, as seen in Fig 2 and S1 Fig, the circular permutation of the sequence does not affect the position of this loop in 3D. To compensate for this in our sequence alignment (Fig 1), the amino acid sequence of PGM3 was modified according to the structural alignment of the proteins (see Methods).

### Sequence preferences of the active site loops

Evolutionary relationships, as well as differences related to substrate preferences or mechanism of the PGM1 paralogs, are presumably reflected in the primary sequence of these proteins. To assess paralog-specific sequence variations, particularly with regard to the active site loops, a multiple sequence alignment was prepared for each of the five proteins. As generalized sequence searches, such as with BLAST [24], do not clearly segregate proteins into the different sub-groups, sequences were manually curated from diverse organisms (Methods), such that pairwise sequence identities within the sub-alignments were less than 90% but more than 40%. The resulting sub-alignments contain 8–17 sequences for each paralog. (The smaller alignments are for PGM2L and PGM5, due to their more restricted evolutionary distribution and high sequence similarity between many vertebrate species.) As placement of sequences into the most appropriate category is not always trivial, even with manual curation, the selected sequence sub-groups were assessed in parallel by phylogenetic analysis (see following section). Sequences used for these analyses are available as S1 File.

**Fig 3:**
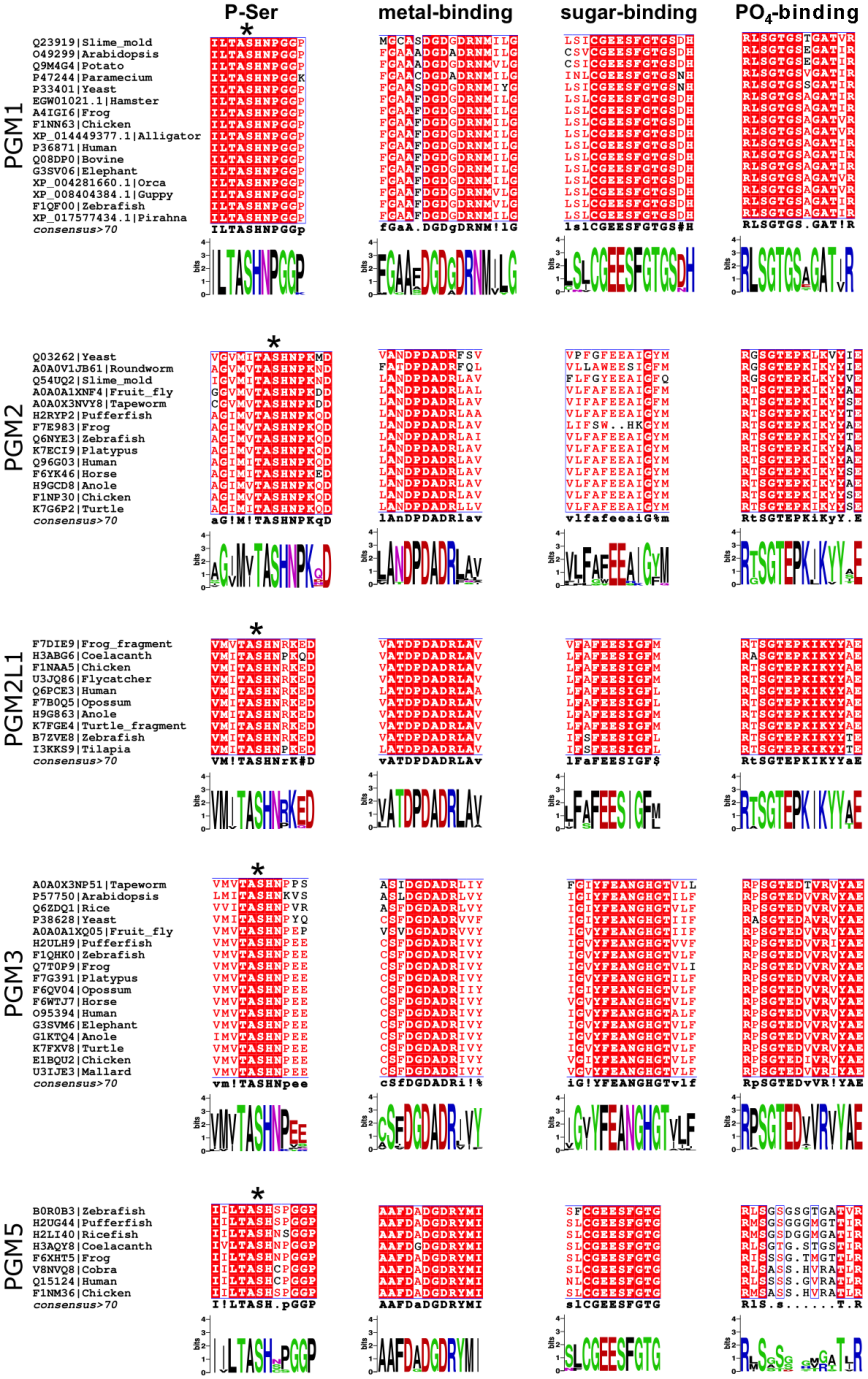
Sequence preferences for key functional regions of the PGM1 paralogs. Selected regions of paralog-specific multiple sequence alignments are shown for the phosphoserine (P-Ser), metal-binding, sugar-binding, and PO_4_-binding loops. Identical residues are highlighted with red background; similar residues are shown in red font. Asterisk (*) indicates the catalytic serine residue.

**Fig 4:**
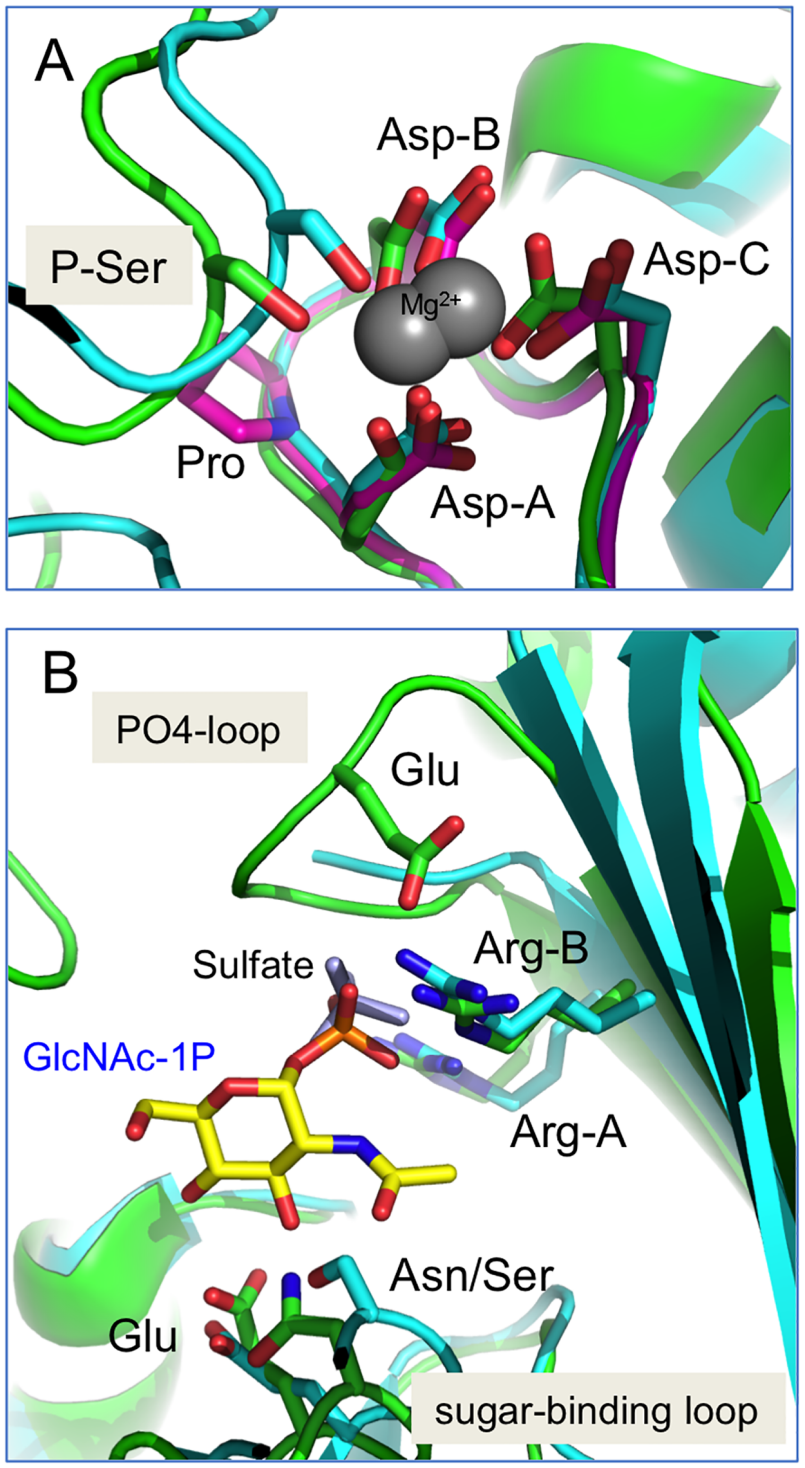
Close-up view of key active site regions of the PGM1 paralogs. **(A)** The catalytic serine and metal-binding loop (regions i- ii) in a superposition of PGM1 (cyan), a PGM3 ortholog (green), and a related bacterial enzyme (PDB ID 2Z0F) (pink). The catalytic serine (shown in its dephospho-state) and the three conserved aspartates (Asp-A,B,C) that coordinate bound metal are highlighted. The bacterial enzyme has a metal-binding loop sequence equivalent to that of PGM2/2L1, and is included to show its structural similarity despite the proline between Asp-A and Asp-B. **(B)** The sugar-binding and PO_4_-binding loops (regions iii-iv) of PGM1 and the PGM3 ortholog. Colors as in (A). The bound substrate (*N*-acetylglucosamine 1-phosphate) from the enzyme-ligand complex in 2DKD is shown in yellow; bound sulfate ion in 5EPC is shown in light blue. In the sugar-binding loop, the conserved glutamate found in all paralogs, and the nearby Ser/Asn (PGM1 vs. PGM3) are labeled. In the PO_4_-binding loop, the two conserved arginines (Arg-A,B) found in PGM1/3/5 are highlighted, along with the conserved glutamate found in PGM2/2L1/3.

The four key regions of the active site described above (i-iv) were analyzed for sequence preferences within each sub-alignment (Fig 3). Regions (i) and (ii) are quite highly conserved in all sub-groups, although some paralog-specific variations are present. In region (i), which contains the catalytic phosphoserine (**bold**), TA**S**H is strictly conserved in all five proteins. However, residues surrounding this motif vary according to sub-group (e.g., TA**S**HNPGG in PGM1 vs. TA**S**HNRKE in PGM2L1). A close-up view of the phosphoserine loop in the active site of PGM1 and PGM3 (Fig 4A) shows correspondingly high structural similarity of this region in these two proteins.

In region (ii), the three aspartates (Asp-A,B,C in Fig 4A) that act as ligands for the metal ion are found in all sequences, but the intervening residues vary according to sub-group. PGM1 has a consensus DGDGDR motif, PGM2/2L1 have DPDADR, while PGM3/5 are more similar to PGM1 (Fig 3). Nevertheless, the structural similarity of the metal-binding loop is retained despite sequence differences (e.g., see pink structure on Fig 4A with a proline between Asp-A and Asp-B). The conserved sequence and structure of regions (i) and (ii) is consistent with their essential functional roles in the enzyme superfamily: phosphoryl transfer and activation of the enzyme by metal binding.

In contrast to regions (i) and (ii), the sequence preference for the sugar-binding loop in region (iii) varies among the paralogs. PGM1/5 contain a GEESF motif, where the first glutamate and the serine (underlined) have known roles in contacting the hydroxyl groups of the sugar [1]. PGM2/2L1 also have two glutamates in their sugar-binding loop, FAFEE, but only PGM2L1 has a serine following these residues. PGM3 has the most distinct motif: YFEAN. This sequence motif retains the conserved, sugar-binding glutamate, but is followed two residues later by an asparagine (rather than a serine). The asparagine makes contacts to the N-acetyl group specific to the substrate of PGM3 [23]. A superposition of the PGM1/3 sugar binding loops is shown in Fig 4B, along with bound substrate N-acetylglucosamine 1-phosphate, from the enzyme-ligand complex of *C*. *albicans* (PDB ID 2DKD). The vicinity of the key Glu and Asn/Ser to the O3 and O4 sugar hydroxyls of the sugar ring is also apparent.

The PO_4_-binding loop (region iv) is a known determinant of substrate binding in the **α**-D-phosphohexomutase superfamily, engaging in multiple interactions with this structurally invariant functional group of the substrate [25]. While generally conserved overall, the sequence of the loop varies somewhat among the paralogs (Fig 3). In PGM1/5, the loop begins and ends with two highly conserved arginines that are predicted to make direct contacts with the phosphate group of the substrate [26]. A sulfate ion is bound to these arginines (Arg-A, B in Fig 4B) in the structure of human PGM1, where it presumably acts as a mimic for the phosphate group of the ligand. In PGM2/2L1, only the first arginine in the loop is conserved, although lysines later in the sequence may serve a similar function. In PGM3, two conserved arginines are also present, although the number of intervening residues between them is smaller than in PGM1. Nevertheless, the two arginines of PGM3 play similar roles in contacting the phosphate group of bound substrate (Fig 4B) [23]. Other residues within the PO_4_-binding loop vary between sub-groups, but often include an SGT or SGS motif, where the serine(s) and threonine also contact the phosphate group of the substrate [23]. A feature distinguishing the PO_4_ –binding loops of PGM2, 2L1, and 3 is a conserved glutamate. In PGM3, this residue appears to help position Arg-B for ligand binding (Fig 4B). The PO_4_ –binding loops of PGM2/2L1 are notable for a conserved proline residue that is not found in the other paralogs.

Based on our updated analysis (Fig 3), and as noted in previous studies, the key active site motifs are quite highly conserved in PGM1 and its paralogs, making these proteins easy to identify as members of the **α**-D-phosphohexomutase superfamily. In the current study, sequence-structure comparisons provide new insights into differences between PGM1 paralogs, with potential mechanistic implications. Using sub-group specific alignments with sequences from a wide range of organisms, we establish paralog-specific sequence differences, which are particularly evident in the sugar-binding and PO_4_-binding loops. These sequence variations should be helpful for categorizing novel sequences into the correct enzyme sub-group (e.g., 2 vs. 2L1) of the superfamily, which is an ongoing challenge in studies of this enzyme family. These sequence preferences also presumably have functional implications. For example, perhaps the lack of a serine in the PGM2 sugar-binding loop (i.e., following the FAFEE motif) is related to the preference of these enzymes for a pentose rather than a hexose-based substrate. Crystal structures, particularly for PGM2/2L1 and their enzyme-ligand complexes, will be necessary to confirm such possibilities. In lieu of structural information, however, the residues identified within these conserved active site motifs are excellent targets for mutagenesis to probe their effects on enzyme activity and substrate preferences, neither of which have been previously investigated in these proteins.

### Phylogenetic relationships

Previous studies have commented on the evolutionary relationships among the various PGM1 paralogs [2–4]. However, these analyses were conducted prior to the current wealth of available genomic data. Moreover, the evolutionary relationships of the human paralogs within the large **α**-D-phosphohexomutase superfamily have not been previously considered. To investigate these issues, a phylogenetic analysis was conducted with MEGA7 [27] using hand-curated groups of sequences for each paralog, as described in the previous section (see also Methods). Selected sequences from distinct branches of the **α**-D-phosphohexomutase superfamily were also included for this analysis. Most of these represent enzymes of known 3D structure [7], and include sequence diverse proteins from bacteria and archaea, as well as sequences from organisms in which these proteins have been functionally studied.

**Fig 5:**
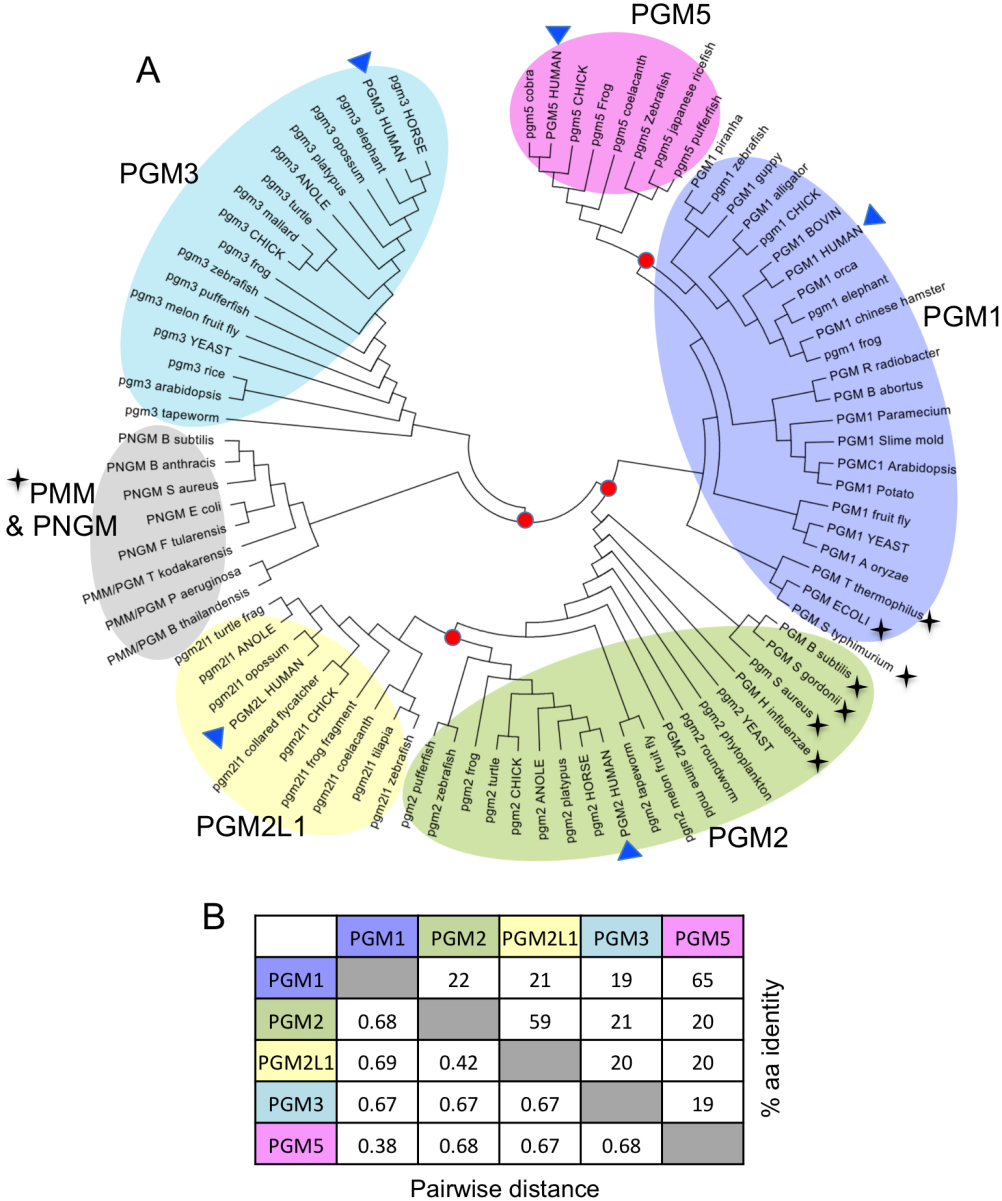
Phylogenetic and sequence relationships among human PGM1 and its paralogs. **(A)** A phylogenetic tree was constructed using MEGA7 [27] to show the evolutionary relationships among the five proteins (see also Methods). Human sequences are highlighted with blue triangles. Bacterial sequences/sub-groups are indicated by black stars. Red circles mark branch points for the emergence of the different paralogs (e.g., between PGM2 and PGM2L1). Selected sequences from two other enzyme sub-groups (PMM & PNGM) within the large **α**-D-phosphohexomutase superfamily are shown as a distinct cluster (gray); these enzymes are found exclusively in bacteria or archaea. **(B)** Amino acid sequence identity matrix for the PGM1 paralogs (sequence references on Table 1). Top-right section of the matrix shows amino acid identities; lower-left shows pairwise sequence distances as calculated by seqinR [28]. Sequence identities with PGM3 were calculated using the altered version of its sequence to account for the circular permutation, as in Fig 1.

A phylogenetic tree (Fig 5A) shows that PGM1 and its paralogs separate clearly into different clades. An early divergence occurred between PGM3 and the other branches of the tree, as well as between PGM1 and PGM2. Both the PGM1 and PGM2 sub-groups are found in organisms from bacteria to vertebrates, while PGM3 appears restricted to eukaryotes. Closer relationships are seen between two pairs of the human paralogs: PGM1 and PGM5, and PGM2 and PGM2L1, suggesting a gene duplication event within their common vertebrate ancestor. This relatively recent divergence is reflected by the relatively high sequence identities between these two pairs of proteins (Fig 5B). On the other hand, the larger evolutionary distance between the other branches (e.g., PGM1 vs. PGM3) is evident from the low sequence identity between these proteins (~20%).

In contrast to the five clades occupied by the human paralogs, other members of the **α**-D-phosphohexomutase superfamily segregate into a distinct evolutionary lineage (gray shading on Fig 5A). These sequences come from two different enzyme sub-groups in the superfamily: phosphomannomutase/phosphoglucomutase (PMM) and phosphoglucosamine mutase (PNGM). (Many thousands of sequences for these enzymes are available; only a few are shown here for comparison). Like PGM1, the PMM proteins can utilize glucose-based phosphosugars as substrates, but can also use mannose phosphosugars, which PGM cannot [29]. The PNGMs are specialized for conversion of glucosamine 1- and 6-phosphate, which is used in the production of UDP-glucosamine and peptidoglycan biosynthesis [30]. Unlike the lineages containing the PGM1 paralogs, these two enzyme sub-groups occur almost exclusively in bacteria or archaea [7,8]. It is not uncommon, however, for microorganisms to have more than one enzyme in the superfamily in their genomes (e.g., PGM2 and PNGM in *Staphylococcus aureus* or PGM1 and PNGM in *E*. *coli*; Fig 5).

In addition to eukaryotic representatives, both the PGM1 and PGM2 lineages contain sequences from bacteria. Based on this limited analysis, it appears that PGM1-like sequences are often found in Gram-negative bacteria, while Gram-positive organisms are found in the PGM2 clade (e.g., the two *Staphylococcus* enzymes). Different sequence motifs in PGM from Gram-positive vs. Gram-negative organisms have been previously noted [31]. This division is not clear-cut, however, as the sequence from Gram-negative *Hemophilus influenza* segregates with PGM2. The presence of PGM2-like bacterial sequences raises the question of when during evolution the functional specialization to a phosphopentomutase occurred. As many bacteria utilize an alternative enzyme family for phosphopentomutase activity [32], it is possible that bacterial PGM2 enzymes are still efficient PGMs. This remains to be investigated.

The phylogenetic relationships and evolutionary distributions of the PGM1 paralogs described here are interesting to consider in light of their known functional roles. For example, an enzyme with efficient PGM activity is likely required for all organisms, consistent with the widespread distribution of PGM1. PGM3 is an addition to the genome of higher organisms, reflecting its role in hexosamine biosynthesis, needed for the production of glycoproteins and other glycoconjugates [33]. PGM2L1 and PGM5 are limited to vertebrates, reflecting their more specialized activities and/or cellular roles. The G16P synthesized by PGM2L1, for instance, has been suggested to have a regulatory role in brain [2], consistent with the vertebrate expression of this enzyme. Similarly, PGM5 has specialized roles in protein-protein interactions in adherens junctions and the Z-disc of cardiomyocytes [5,6,18,34]. Overall, our findings emphasize both the ancient evolutionary history of the **α**-D-phosphohexomutase superfamily, as well as the continuing adaptation of these enzymes to support specialized functional roles in higher organisms.

### Tissue-specific expression

**Fig 6:**
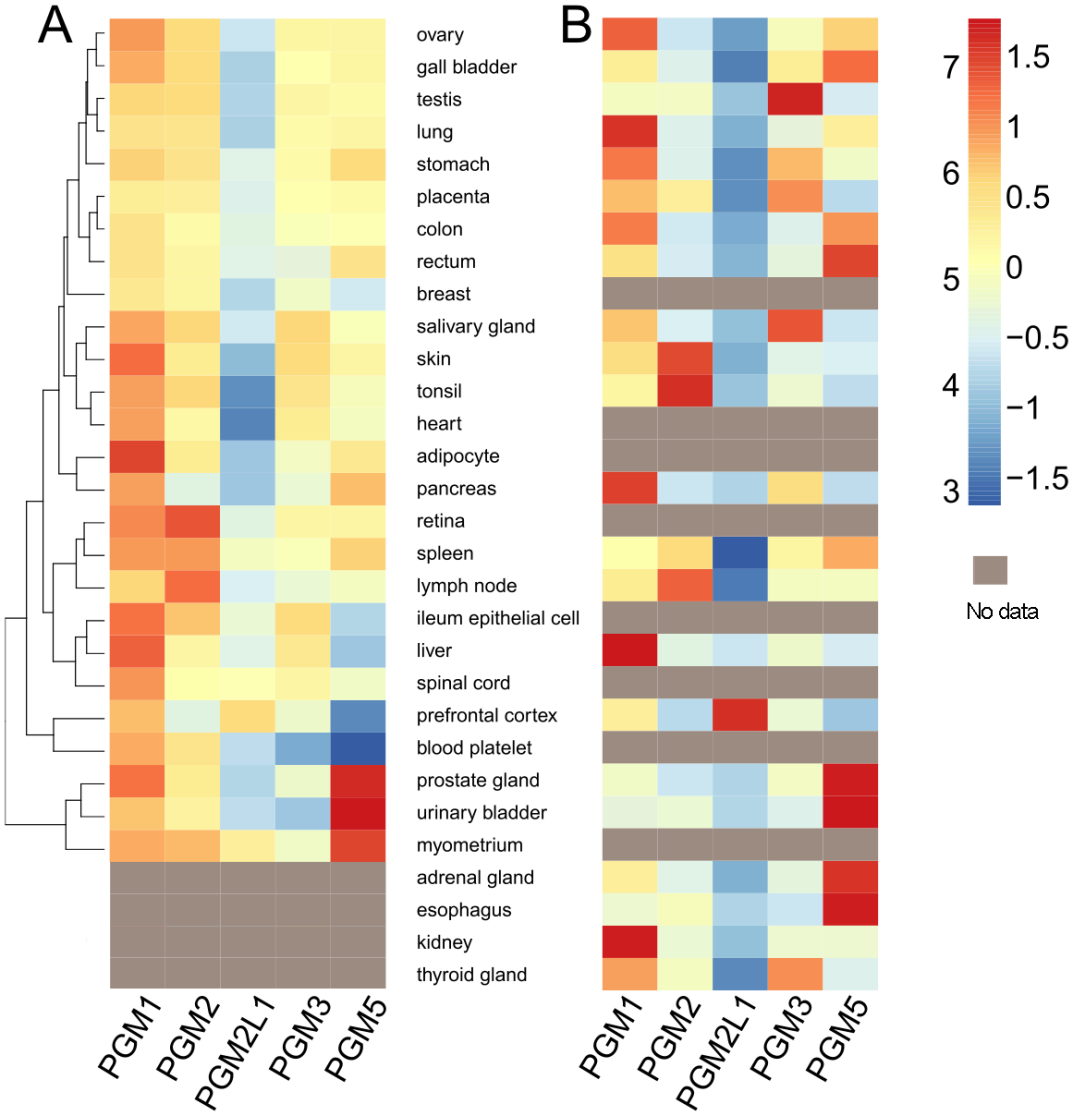
Tissue-specific protein expression profiles of the PGM1 paralogs. Data are from ProteomicsDB [35] for 30 human tissues. (**A)** Protein expression levels are shown on a log scale using their iBAQ scores. Brackets at left indicate clustering of tissues into groups with similar protein expression patterns. (**B)** RNA expression levels. Data are normalized across each row by Z-score, emphasizing the relative RNA expression for the paralogs in each tissue. (Z-score is the standard deviation from the mean per row). Gray cells indicate that the corresponding tissues are missing in the respective database.

Several public databases were examined for expression data on PGM1 and its four paralogs. These databases contain a wealth of RNA and protein expression data on these five proteins that well exceeds that in published studies. Among the available databases, ProteomicsDB [35] (https://www.proteomicsdb.org/) was found to be quite comprehensive and generally representative; for an alternate resource, see S2 Fig. Protein and RNA expression data for 30 human tissues were downloaded from ProteomicsDB and visualized as a heat map (Fig 6). As the un-scaled RNA data are dominated by extremely high levels of PGM5 in esophagus, the RNA scores (Fig 6B) were normalized across each row (per tissue). Consequently, RNA data within a column cannot be directly compared due to different scales, while protein data can be compared across both rows and columns.

For both protein and RNA expression, PGM1 shows the highest expression levels in the greatest number of tissues, consistent with its critical role in primary metabolism (Fig 6). Relatively high expression levels are found in liver (both protein and RNA), adipocytes (protein), and kidney/lung (RNA), with intermediate expression in most other tissues. High expression of PGM1 in liver and skeletal muscle is well known [20]; skeletal muscle data is not present in Proteomics DB, but is included on S2 Fig, where intermediate expression is seen.

Expression of PGM2 is also fairly widespread in human tissues (Fig 6). Protein expression is highest in lymph nodes, retina and spleen, with moderate expression in most other tissues. Relative RNA expression is highest in tonsil, skin, and lymph nodes. These results are consistent with published data showing high-moderate RNA expression for PGM2 in a number of tissues in mice [2]. In contrast to PGM2, PGM2L1 appears to be well expressed in only a few tissues. Relatively high protein/RNA expression is found in the prefrontal cortex (brain); many other tissues have markedly low levels. Previous studies [2] in mice also indicated high levels in brain; however, high levels in testis noted in this earlier study are not confirmed here.

Human PGM3 shows moderate expression in many tissues. RNA expression is relatively high in testis, salivary gland, thyroid, and placenta (Fig 6B). This generally agrees with published data [36], which also noted relatively high mRNA levels in placenta, as well as in heart, liver, and pancreas. Protein expression (Fig 6A) is moderate overall, with intermediate levels in salivary gland, ileum epithelia, and skin, and other tissues. It has been previously suggested that expression levels of PGM3 correlate with the levels of glycosylated proteins produced by the respective tissues [36]; data in Fig 6 support this, showing high PGM3 expression in many tissues with secretory functions.

PGM5, also known as aciculin, had been previously noted for its protein expression in smooth, skeletal, and cardiac muscle [4,5,18,34]. Here, we find the highest protein expression levels of PGM5 occurs in tissues rich in smooth muscle, including prostate, bladder, and uterus (myometrium), with moderate levels found in the pancreas, spleen, rectum, and stomach. As noted earlier, RNA expression of PGM5 in esophagus dwarfs that of all other tissues, consistent with the muscular enrichment of this organ. Relatively high RNA levels for PGM5 are also seen in the spleen, gall bladder, and adrenal gland.

The data presented here are the first side-by-side comparison of tissue-specific expression patterns of the PGM1 paralogs, across a wide array of tissue types and assessed with the same methodology. As described above, this information provides a new appreciation of their biological functions, highlighting the tissues and organs that benefit from their respective enzymatic activities and/or structural roles. Expression patterns are also critical for understanding the possibility of functional overlap among these proteins, particularly with regard to their phosphoglucomutase activity. For example, while PGM2 and PGM3 have preferences for substrates containing sugars other than glucose, both of them can also catalyze the interconversion of glucose 1-phosphate and glucose 6-phosphate, albeit less effectively [2,15]. The possibility of functional substitution of one paralog for another is highly relevant for understanding disease phenotypes associated with deficiencies of these proteins. For example, in PGM1 deficiency, it is not understood how some patients can lead relatively normal lives [9,37,38], given the critical role of this enzyme in glucose metabolism. One possibility for this may be functional overlap with the PGM1 paralogs, at least in certain tissues. In particular, the presence of the high levels of PGM2L1 in brain has been suggested to limit the cognitive impacts of PGM1 deficiency [9].

### Identification of potential disease-causing missense variants

**Fig 7:**
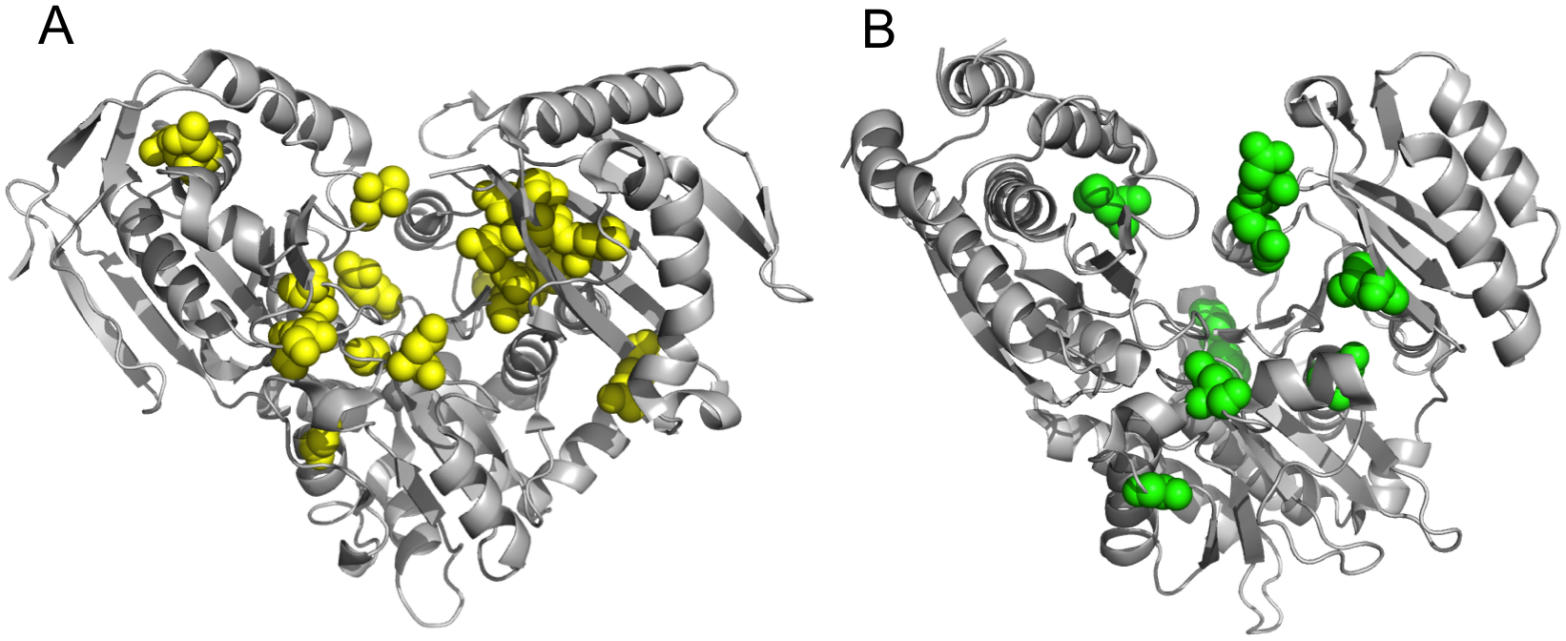
Residues affected by disease-related mutants mapped onto the structures of (A) PGM1 (PDB ID 5EPC) and (B) a homolog of PGM3 (PDB ID 2DKD). Residues affected by PGM1 missense variants are highlighted in yellow; those in PGM3 in green.

Enzyme deficiencies of both PGM1 and PGM3 are associated with inherited disease in humans [9–14]. Currently, 18 residue positions in PGM1 and eight in PGM3 have established disease-related variants. These residues are highlighted on Fig 1 (gold and cyan triangles) and Fig 7. These missense mutations are found throughout the protein sequence, including within key areas of the active site (blue shading). This is most evident in the case of the PO_4_-binding loop (region iv) in the C-terminal domain of the protein, where both PGM1 and PGM3 have multiple known missense variants. In the case of PGM1, a number of missense variants have also been associated with interdomain interfaces of the enzyme [26].

Given the recent identification of disease-related mutations for PGM1 and PGM3, it seems opportune to investigate whether detrimental variants for the other paralogs may occur in the human population. While such variants have not yet been identified in patients, it seems likely that they exist [2], and their prediction should be feasible given the sequence-structure relationships of the paralogs, and the available structural models for PGM1 and PGM3. Moreover, evolutionary constraint scores from ExAc (reflecting intolerance to genetic variation) are notably high for both PGM2L1 and PGM5, suggesting that disease-related mutations are a likely possibility in these proteins (S1 Table).

As our source for genetic variants, we utilized the Exome Aggregation Consortium (ExAC) database, which comprises genomic information from ~60,000 healthy individuals. As many inherited diseases (including PGM1 and PGM3 deficiency) are autosomal recessive, individuals with a detrimental change in only one allele are healthy, and their sequences would still be included in the database. This notion was confirmed by identification of several known PGM1 missense variants in the ExAC database (i.e., G121R, D263Y, G291R, G330, R422W, and R503Q).

Sequence variants for each of the PGM1 paralogs were examined in ExAC, and those with a likely detrimental impact on function compiled (Table 2). Rather than using computational algorithms, which give rather non-specific predictions for the paralogs due to the high sequence conservation within sub-groups, we adopted a knowledge-based approach for selecting ExAc variants likely to cause protein dysfunction. Selection was based on prior information of disease-related variants and/or knowledge of enzyme function, as follows: 1) variants that directly correspond to residue positions associated with disease in either PGM1 or PGM3; or 2) variants of residues found within key regions of the active site. In both cases, only substitutions that produce a significant physicochemical change of the residue (e.g., G→R) were included. We then validated our predicted disease-related variants with PolyPhen-2 [39], which confirmed 54 of the 64 originally selected; the remaining ten were discarded. Finally, as high frequency mutations are unlikely to be disease-related, the frequency of each variant in the human population was evaluated (as annotated in ExAc). All variants were found to be rare (Table 2), with most being singletons.

**Table 2:** Known and predicted disease-associated missense variants in the PGM1 paralogs.

	PGM1	PGM2	PGM2L1	PGM3	PGM5
**Domain 1**					
*Patient variants*:	T19A				
	N38Y			R41L/H*	
	Q41R(E/H)				
	D62H				
	T115A				
	G121R(W)				
	A139T			L83S	
*Functional region variants*:				A63V	
		S165P			
		H166D			
			R178C*		
**Domain 2**					
*Patient variants*:	G230E				
				D239H	
	H261D		Q291K	N246S	
	D263G/Y				
	G291R				
*Functional region variants*:	D288G	D322N*/E	D332V		
		P323L^			
		D324H/G	D334E*		D295V*
**Domain 3**					
*Patient variants*:				D297E	
	G330R(S*)				
	P336R				
	T337M				
	E377K		E435G		
				F379L	
	E388K				
*Functional region variants*:			F431L		
			A432E		
	G375V*		F433S		
				F369I	
					S383R
			I437T*	N372Y	
	G380R*	G428E			
					T386I
**Domain 4**					
*Patient variants*:	R422W(Q)				R427C/H*
			C520Y	Q451R	
	R503Q	R558C*/H^		R496W*	R508W*/Q*
	G508R	E563V		E501Q(G)	S513R^
				D502Y	
	R515L/Q(W*)			R505Q	
	L516P				
*Functional region variants*:	S505R				
	G506S		G571R*		
	G511R*				R516W*

Variants are tabulated according to protein domain (1–4) and by association with either patient mutations (top sub-sections) or key functional regions (bottom sub-sections). Matching residue positions for the five proteins were determined using the sequence alignment on Fig 1 and a 3D structural alignment (PDB IDs 5EPC and 2DKD). Confirmed disease-related variants of PGM1 and PGM3 are highlighted with red shading; predicted variants in ExAC that correspond to these are in yellow. Predicted dysfunctional variants occurring at the same residue position as a known disease-related variant are shown in parentheses. All missense variants from ExAc are singletons (found in a single individual; unmarked) or present at very low frequencies as marked: <1/10000 (*) or between 1/10000–0.001 (^).

In the first category described above, there are 26 residue positions with known missense variants in either PGM1 or PGM3 (red shading on Table 2). In ExAC, we find 18 additional variants at corresponding positions in the paralogs that appear likely to cause dysfunction (black stars on Fig 1; yellow shading on Table 2). The second category includes ~35 residue positions (blue regions on Fig 1). Using the criteria above, we find 29 variants at 27 residue positions of the paralogs that fit into this category (Table 2, unshaded residues).

Using our combined, knowledge-based approach, we conservatively identify 16 additional sequence variants in PGM1 and PGM3, and eleven more in PGM2, PGM2L1, and PGM5 that appear likely to cause protein dysfunction (i.e., impaired folding or catalysis). These occur in all four domains of the protein, and include the active site loops. The conserved PO_4_-binding loop of domain 4, in particular, appears to be enriched with likely candidates. For example, in the case of the missense variant R503Q associated with PGM1 deficiency, we find corresponding variants in PGM2 (R558C/H), PGM3 (R496W), and PGM5 (R508W/Q), all of which show a similar and significant physicochemical change to this key PO_4_-binding residue [1]. In contrast, corresponding disease-associated variants among the paralogs are sparse in domain 3, which includes the sugar-binding loop. This is likely due to the paralog-specific sequence differences in this region, discussed above, which makes the identification of key functional residues more challenging.

Although all the PGM1 paralogs have potential disease-related variants in the ExAC database, in the case of PGM5, several caveats should be noted. On one hand, residues in PGM5 that correspond to disease-associated mutations in PGM1 should be quite reliable, given the significant amino acid identity between these two proteins (Fig 5B). On the other hand, as PGM5 is proposed to play a structural rather than enzymatic role in the cell [4], it is unclear whether the proposed variants will also affect this function. Missense variants that impair protein folding, which account for ~50% of those known in PGM1 [40], would likely still impact the function of PGM5. However, this might not be the case for mutants that are exclusively catalytic in nature (e.g., D263G/Y of PGM1) [41]. Experimental studies that define regions of functional importance in PGM5 will be needed to resolve these issues.

## Conclusions

Human PGM1 and its paralogs share a long and intertwined evolutionary history that is evident from the sequence conservation of key functional regions and similar 3D topologies. These interconnections make it difficult to fully appreciate the varying biochemical functions and tissue-specific impacts of these proteins, when considered solely on an individual basis. The current study provides, for the first time, a side-by-side analysis of sequence, structure, evolution, and expression patterns for all five of these related proteins. This is a critical step toward disentangling the individual roles and relative importance of these proteins in cellular biology and human disease. This work also establishes a baseline for future experimental studies, such as the design of site-directed mutants to probe enzyme function. In combination with biochemical data on enzyme activity, tissue-specific expression patterns may provide insights into the complex disease phenotypes in PGM1 and PGM3 deficiencies. Finally, our analysis of variants currently present in the human population suggests that inherited disorders of PGM2/2L1/5 may be identified.

## Methods

### Sequence alignment of the PGM1 paralogs

A multiple sequence alignment was created using MUSCLE 3.8 [42] and the protein sequences from UniProt KB (Table 1). (For treatment of the circular permutation in PGM3, see following paragraph.) The alignment was formatted with ESPript 3.0 [43]. Pairwise sequence identities were calculated by MUSCLE 3.8 [42].

To compensate for a circular permutation in human PGM3 [23], the amino acid sequence of this protein was adjusted to permit maximal alignment of conserved regions in domain 1. To accomplish this, a structural alignment of human PGM1 (PDB ID 5EPC) and an ortholog of human PGM3 (PDB ID 2DKA) from C. *albicans* was generated using TOPP from the CCP4 program package [44]. Structurally conserved elements were identified on a pairwise sequence alignment of PGM3 and 2DKA (49% identity) made with Clustal Omega [45]. Sequence segments that corresponded to the structurally conserved elements were manually rearranged in the sequence of PGM3 to match those of PGM1 (i.e., residues 54–114 of human PGM3 were swapped with residues 115–169; see Fig 1 and S1 Fig).

### Sequence analysis of active site motifs

Individual multiple sequence alignments for each of the paralogs were compiled using diverse sequences hand curated from UniProt, and were aligned using Clustal Omega 1.2.2 [45] and formatted with Espript 3.0 [43]. Sequence preferences for key active site regions are displayed using WebLogo [46]. Within each sub-alignment, pairwise sequence identities were typically ~40–90%; alignments for the PGM2L and PGM5 proteins contain fewer sequences and/or are less diverse due to their exclusively vertebrate lineage.

### Phylogenetic analyses

Evolutionary analyses of the PGM1 paralogs were conducted with MEGA7 [27] using the sequences from the sub-alignments described above. Additional sequences for other members of the enzyme superfamily were added as described in text. The evolutionary history was inferred using the maximum likelihood method based on the JTT matrix-based model [47]. Initial tree(s) for the heuristic search were obtained automatically by applying Neighbor-Join and BioNJ algorithms to a matrix of pairwise distances estimated using a JTT model, and selecting the topology with the superior log likelihood value. The analysis involved 85 amino acid sequences. All positions containing gaps and missing data were eliminated. There were a total of 265 positions in the final dataset. The tree with the highest log likelihood (-23090.0265) is shown. Sequences used for these analyses are available as S1 File.

### Tissue-specific expression

Protein and mRNA expression data were downloaded from the ProteomicsDB (https://www.proteomicsdb.org/). Proteomic and RNA-Seq information in this database was compiled from publicly available and specifically generated data, as described in [35]. Protein expression levels of the PGM1 paralogs were normalized using the intensity-based absolute-protein-quantification method (iBAQ) from the ProteomicsDB. The iBAQ scores were plotted as a heatmap using the Nonnegative Matrix Factorization R-package [46]. RNA data in fragments per kilobase of transcript per million were normalized and presented with per-row Z-scoring (standard deviation from the mean). Only tissue expression data were used; fluid expression levels were excluded. Tissues where no expression was observed for any of the paralogs were not included in our analyses. Data used in this study are available in S2 File. Protein expression data for the PGM1 paralogs from an alternate resource, the Human Protein Atlas [48] can be found in S2 Fig.

### Prediction of disease-related variants

Potential disease-related variants of the PGM1 paralogs were selected from among confirmed sequence variants in the human population compiled in the ExAC database. Transcripts from ExAC used were: ENST00000371084 (PGM1), ENST00000381967 (PGM2), ENST00000298198 (PGM2L1), ENST00000513973 (PGM3), and ENST00000396396 (PGM5). Number of variants in ExAC for each paralog ranged from >200 for PGM1 and PGM2, to ~130–150 for the others. Based on the multiple sequence alignment (Fig 1) and a 3D structural alignment (PDB IDs 5EPC and 2DKD), variants that corresponded to residues affected by missense mutations of PGM1 or PGM3, or those impacting key functional regions (Fig 1), were tabulated (Table 2). In both categories, only variants producing significant physicochemical changes were selected. All hand-selected variants were subsequently screened with PolyPhen-2 [39].

## Supporting information

S1 Fig Details of the sequence modification of human PGM3 for multiple sequence alignments. **(A)** Sequences of wild-type human PGM3 and the modified version used for sequence alignments, showing transposed regions (see text). Residues highlighted in magenta were swapped with those in green. **(B)** Superposition of human PGM1 (white) and the PGM3 homolog from *C*. *albicans* (PDB ID 2DKA) (pink). Green/magenta highlight the regions affected by circular permutation (and swapped in the amino acid sequence), showing how they are structurally conserved despite the different sequence connectivity in PGM3.
(PDF)

S2 Fig Protein expression data for the PGM1 paralogs from the Human Protein Atlas [48] showing data for a wide range of tissues. Data are binned into low (light blue), medium (orange), or high (red) expression. Dark blue indicates no measureable expression; tissues with no expression for any paralog were omitted.
(PDF)

S1 File Fasta sequence files used in phylogenetic analysis. (TXT)

S2 File Tissue-specific expression data for the PGM1 paralogs obtained from ProteomicsDB. (XLSX)

S1 Table No. of expected vs. observed variants of the PGM1 paralogs and their evolutionary constraint scores. (PDF)

## References

[1] Beamer LJ. Mutations in hereditary phosphoglucomutase 1 deficiency map to key regions of enzyme structure and function. J Inherit Metab Dis. 2015;38. 10.1007/s10545-014-9757-9 25168163

[2] Maliekal P, Sokolova T, Vertommen D, Veiga-da-Cunha M, Van Schaftingen E. Molecular identification of mammalian phosphopentomutase and glucose-1,6-bisphosphate synthase, two members of the α-D- phosphohexomutase family. J Biol Chem. 2007;282: 31844–31851. doi: 10.1074/jbc.M706818200 17804405 10.1074/jbc.M706818200

[3] Mio T, Yamada-Okabe T, Arisawa M, Yamada-Okabe H. Functional cloning and mutational analysis of the human cDNA for phosphoacetylglucosamine mutase: Identification of the amino acid residues essential for the catalysis. Biochim Biophys Acta. 2000;1492: 369–376. 11004509 10.1016/s0167-4781(00)00120-2

[4] Moiseeva E, Belkin A, Spurr N, Koteliansky V, Critchley D. A novel dystrophidutrophin-associatedproteinis an enzymatically inactive member of the phosphoglucomutase superfamily. Eur J Biochem. 2004;235: 103–113. 10.1111/j.1432-1033.1996.00103.x 8631316

[5] Molt S, Bührdel JB, Yakovlev S, Schein P, Orfanos Z, Kirfel G, et al Aciculin interacts with filamin C and Xin and is essential for myofibril assembly, remodeling and maintenance. J Cell Sci. 2014; 3578–3592. doi: 10.1242/jcs.152157 24963132 10.1242/jcs.152157

[6] Belkin AM, Burridge K. Association of aciculin with dystrophin and utrophin. J. Biol. Chem. 1995 pp. 6328–6337. 7890770 10.1074/jbc.270.11.6328

[7] Stiers KM, Muenks AG, Beamer LJ. Biology, mechanism, and structure of enzymes in the α-D-phosphohexomutase superfamily In: Karabenchova-Christova T, editor. Advances in Protein Chemistry and Structural Biology. Burlington: Academic Press; 2017 pp. 265–304. doi: 10.1016/bs.apcsb.2017.04.005 10.1016/bs.apcsb.2017.04.005 PMC5802415 28683921

[8] Shackelford GS, Regni CA, Beamer LJ. Evolutionary trace analysis of the alpha-D-phosphohexomutase superfamily. Protein Sci. 2004;13: 2130–2138. doi: 10.1110/ps.04801104 15238632 10.1110/ps.04801104 PMC2279825

[9] Tegtmeyer LC, Rust S, van Scherpenzeel M, Ng BG, Losfeld M-E, Timal S, et al Multiple Phenotypes in Phosphoglucomutase 1 Deficiency. N Engl J Med. 2014;370: 533–542. doi: 10.1056/NEJMoa1206605 24499211 10.1056/NEJMoa1206605 PMC4373661

[10] Sassi A, Lazaroski S, Wu G, Haslam SM, Fliegauf M, Mellouli F, et al Hypomorphic homozygous mutations in phosphoglucomutase 3 (PGM3) impair immunity and increase serum IgE levels. J Allergy Clin Immunol. 2014;133: 1410–9, 1419–13. doi: 10.1016/j.jaci.2014.02.025 24698316 10.1016/j.jaci.2014.02.025 PMC4825677

[11] Lundin KE, Hamasy A, Backe PH, Moens LN, Falk-Sörqvist E, Elgstøen KB, et al Susceptibility to infections, without concomitant hyper-IgE, reported in 1976, is caused by hypomorphic mutation in the phosphoglucomutase 3 (PGM3) gene. Clin Immunol. 2015;161: 366–372. doi: 10.1016/j.clim.2015.10.002 26482871 10.1016/j.clim.2015.10.002 PMC4695917

[12] Stray-Pedersen A, Backe PH, Sorte HS, Mørkrid L, Chokshi NY, Erichsen HC, et al PGM3 mutations cause a congenital disorder of glycosylation with severe immunodeficiency and skeletal dysplasia. Am J Hum Genet. 2014;95: 96–107. doi: 10.1016/j.ajhg.2014.05.007 24931394 10.1016/j.ajhg.2014.05.007 PMC4085583

[13] Zhang Y, Yu X, Ichikawa M, Lyons JJ, Datta S, Lamborn IT, et al Autosomal recessive phosphoglucomutase 3 (PGM3) mutations link glycosylation defects to atopy, immune deficiency, autoimmunity, and neurocognitive impairment. J Allergy Clin Immunol. 2014;133: 1400–1409. doi: 10.1016/j.jaci.2014.02.013 24589341 10.1016/j.jaci.2014.02.013 PMC4016982

[14] Pacheco-Cuéllar G, Gauthier J, Désilets V, Lachance C, Lemire-Girard M, Rypens F, et al A novel PGM3 mutation is associated with a severe phenotype of bone marrow failure, severe combined immunodeficiency, skeletal dysplasia, and congenital malformations. J Bone Miner Res. 2017;in press. doi: 10.1002/jbmr.3173 28543917 10.1002/jbmr.3173

[15] McAlpine PJ, Hopkinson DA, Harris H. The relative activities attributable to the three phosphoglucomutase loci (PGM1, PGM2, PGM3) in human tissues. Ann Hum Genet. 1970;34: 169–175. 5493845 10.1111/j.1469-1809.1970.tb00230.x

[16] Quick CB, Fisher RA, Harris H. A Kinetic Study of the Isozymes Determined by the Three Human Phosphoglucomutase Loci PGM1, PGM2 and PGM3. Eur J Biochem. 1974;42: 511–517. 4829444 10.1111/j.1432-1033.1974.tb03366.x

[17] Pang H, Koda Y, Soejima M, Kimura H. Identification of human phosphoglucomutase 3 (PGM3) as N-acetylglucosamine-phosphate mutase (AGM1). Ann Hum Genet. 2002;66: 139–44. doi: 10.1017/S0003480002001033 12174217 10.1017/S0003480002001033

[18] Belkin AM, Klimanskaya I V, Lukashev ME, Lilley K, Critchley DR, Koteliansky VE. A novel phosphoglucomutase-related protein is concentrated in adherens junctions of muscle and nonmuscle cells. J Cell Sci. 1994;107: 159–173. 8175905 10.1242/jcs.107.1.159

[19] Gaudet P, Livstone MS, Lewis SE, Thomas PD. Phylogenetic-based propagation of functional annotations within the Gene Ontology consortium. Brief Bioinform. 2011;12: 449–462. doi: 10.1093/bib/bbr042 21873635 10.1093/bib/bbr042 PMC3178059

[20] Putt W, Ives JH, Hollyoake M, Hopkinson DA, Whitehouse DB, Edwards YH. Phosphoglucomutase 1: a gene with two promoters and a duplicated first exon. Biochem J. 1993;296: 417–22. 8257433 10.1042/bj2960417 PMC1137712

[21] Gururaj A, Barnes CJ, Vadlamudi RK, Kumar R. Regulation of phosphoglucomutase 1 phosphorylation and activity by a signaling kinase. Oncogene. 2004;23: 8118–27. doi: 10.1038/sj.onc.1207969 15378030 10.1038/sj.onc.1207969

[22] Arimura T, Inagaki N, Hayashi T, Shichi D, Sato A, Hinohara K, et al Impaired binding of ZASP/Cypher with phosphoglucomutase 1 is associated with dilated cardiomyopathy. Cardiovasc Res. Oxford University Press; 2009;83: 80–88. 10.1093/cvr/cvp119 19377068

[23] Nishitani Y, Maruyama D, Nonaka T, Kita A, Fukami TA, Mio T, et al Crystal structures of N-acetylglucosamine-phosphate mutase, a member of the a-D-phosphohexomutase superfamily, and its substrate and product complexes. J Biol Chem. 2006;281: 19740–19747. doi: 10.1074/jbc.M600801200 16651269 10.1074/jbc.M600801200

[24] Altschul SF, Madden TL, Schäffer AA, Zhang J, Zhang Z, Miller W, et al Gapped BLAST and PSI-BLAST: A new generation of protein database search programs. Nucleic Acids Research. 1997 pp. 3389–3402. doi: 10.1093/nar/25.17.3389 9254694 10.1093/nar/25.17.3389 PMC146917

[25] Regni C, Naught L, Tipton PA, Beamer LJ. Structural Basis of Diverse Substrate Recognition by the Enzyme PMM/PGM from P. aeruginosa. Structure. 2004;12: 55–63. 14725765 10.1016/j.str.2003.11.015

[26] Stiers KM, Kain BN, Graham AC, Beamer LJ. Induced structural disorder as a molecular mechanism for enzyme dysfunction in phosphoglucomutase 1 deficiency. J Mol Biol. 2016;428: 1493–1505. doi: 10.1016/j.jmb.2016.02.032 26972339 10.1016/j.jmb.2016.02.032 PMC5802404

[27] Kumar S, Stecher G, Tamura K. MEGA7: Molecular Evolutionary Genetics Analysis version 7.0 for bigger datasets. Mol Biol Evol. 2016; msw054. 10.1093/molbev/msw054 PMC8210823 27004904

[28] Charif D, Lobry J, Necsulea A, Palmeira L, Penel MS. The seqinr Package. R Packag. 2007;

[29] Lowry OH, Passonneau JV. Phospoglucomutase kinetics with the phosphates of fructose, glucose, mannose, ribose, and galactose. Biochemistry. 1969;244: 910–916. 5769188

[30] Barreteau H, Kovac A, Boniface A, Sova M, Gobec S, Blanot D. Cytoplasmic steps of peptidoglycan biosynthesis. FEMS Microbiol Rev. 2008;32: 168–207. doi: 10.1111/j.1574-6976.2008.00104.x 18266853 10.1111/j.1574-6976.2008.00104.x

[31] Mehra-Chaudhary R, Mick J, Tanner JJ, Henzl MT, Beamer LJ. Crystal structure of a bacterial phosphoglucomutase, an enzyme involved in the virulence of multiple human pathogens. Proteins. 2011;79: 1215–29. doi: 10.1002/prot.22957 21246636 10.1002/prot.22957 PMC3066478

[32] Galperin MY, Koonin E V, Bairoch A. A superfamily of metalloenzymes unifies phosphopentomutase and cofactor-independent phosphoglycerate mutase with alkaline phosphatases and sulfatases. Protein Sci. 1998;7: 1829–1835. doi: 10.1002/pro.5560070819 10082381 10.1002/pro.5560070819 PMC2144072

[33] Willems AP, van Engelen BGM, Lefeber DJ. Genetic defects in the hexosamine and sialic acid biosynthesis pathway. Biochim Biophys Acta. 2016;1860: 1640–54. doi: 10.1016/j.bbagen.2015.12.017 26721333 10.1016/j.bbagen.2015.12.017

[34] Belkin AM, Burridge K. Expression and localization of the phosphoglucomutase-related cytoskeletal protein, aciculin, in skeletal muscle. J Cell Sci. 1994;107: 1993–2003. 7983164 10.1242/jcs.107.7.1993

[35] Wilhelm M, Schlegl J, Hahne H, Gholami AM, Lieberenz M, Savitski MM, et al Mass-spectrometry-based draft of the human proteome. Nature. Nature Publishing Group; 2014;509: 582–587. doi: 10.1038/nature13319 24870543 10.1038/nature13319

[36] Li C, Rodriguez M, Banerjee D. Cloning and characterization of complementary DNA encoding human N-acetylglucosamine-phosphate mutase protein. Gene. 2000;242: 97–103. 10721701 10.1016/s0378-1119(99)00543-0

[37] Stojkovic T, Vissing J, Petit F, Piraud M, Orngreen MC, Andersen G, et al Muscle Glycogenosis Due to Phosphoglucomutase 1 Deficiency. N Engl J Med. 2009;361: 425–427. doi: 10.1056/NEJMc0901158 19625727 10.1056/NEJMc0901158

[38] Pérez B, Medrano C, Ecay MJ, Ruiz-Sala P, Martínez-Pardo M, Ugarte M, et al A novel congenital disorder of glycosylation type without central nervous system involvement caused by mutations in the phosphoglucomutase 1 gene. J Inherit Metab Dis. 2013;36: 535–542. doi: 10.1007/s10545-012-9525-7 22976764 10.1007/s10545-012-9525-7

[39] Adzhubei I, Jordan DM, Sunyaev SR. Predicting Functional Effect of Human Missense Mutations Using PolyPhen-2. Current Protocols in Human Genetics. 2001 p. Unit7 20 doi: 10.1002/0471142905.hg0720s76 23315928 10.1002/0471142905.hg0720s76 PMC4480630

[40] Lee Y, Stiers KM, Kain BN, Beamer LJ. Compromised catalysis and potential folding defects in in vitro studies of missense mutants associated with hereditary phosphoglucomutase 1 deficiency. J Biol Chem. 2014;289: 32010–32019. doi: 10.1074/jbc.M114.597914 25288802 10.1074/jbc.M114.597914 PMC4231678

[41] Stiers KM, Graham AC, Kain BN, Beamer LJ. Asp263 missense variants perturb the active site of human phosphoglucomutase 1 (PGM1). FEBS J. 2017;284: 937–947. doi: 10.1111/febs.14025 28117557 10.1111/febs.14025 PMC5802412

[42] Edgar RC. MUSCLE: Multiple sequence alignment with high accuracy and high throughput. Nucleic Acids Res. 2004;32: 1792–1797. doi: 10.1093/nar/gkh340 15034147 10.1093/nar/gkh340 PMC390337

[43] Robert X, Gouet P. Deciphering key features in protein structures with the new ENDscript server. Nucleic Acids Res. 2014;42. 10.1093/nar/gku316 PMC4086106 24753421

[44] Winn MD, Ballard CC, Cowtan KD, Dodson EJ, Emsley P, Evans PR, et al Overview of the CCP4 suite and current developments. Acta Crystallographica Section D: Biological Crystallography. 2011 pp. 235–242. doi: 10.1107/S0907444910045749 21460441 10.1107/S0907444910045749 PMC3069738

[45] Sievers F, Wilm A, Dineen D, Gibson TJ, Karplus K, Li W, et al Fast, scalable generation of high-quality protein multiple sequence alignments using Clustal Omega. Mol Syst Biol. 2011;7: 539 doi: 10.1038/msb.2011.75 21988835 10.1038/msb.2011.75 PMC3261699

[46] Crooks GE, Hon G, Chandonia JM, Brenner SE. WebLogo: A sequence logo generator. Genome Res. 2004;14: 1188–1190. doi: 10.1101/gr.849004 15173120 10.1101/gr.849004 PMC419797

[47] Jones DT, Taylor WR, Thornton JM. The rapid generation of mutation data matrices from protein sequences. Bioinformatics. 1992;8: 275–282. 10.1093/bioinformatics/8.3.275 1633570

[48] Uhlen M, Fagerberg L, Hallstrom BM, Lindskog C, Oksvold P, Mardinoglu A, et al Tissue-based map of the human proteome. Science (80-). 2015;347: 1260419–1260419. doi: 10.1126/science.1260419 25613900 10.1126/science.1260419

