# Supplementary material for: Sequence-structure relationships, expression profiles, and disease-associated mutations in the paralogs of phosphoglucomutase 1
Source: PLoS One. 2017 Aug 24;12(8):e0183563. doi: 10.1371/journal.pone.0183563 (PMC5570346; doi:10.1371/journal.pone.0183563)
Supplement: S1 Fig — (A) Sequences of wild-type human PGM3 and the modified version used for sequence alignments, showing transposed regions (see text). Residues highlighted in magenta were swapped with those in green. (B) Superposition of human PGM1 (white) and the PGM3 homolog from C. albicans (PDB ID 2DKA) (pink). Green/magenta highlight the regions affected by circular permutation (and swapped in the amino acid sequence), showing how they are structurally conserved despite the different sequence connectivity in PGM3. (PDF) [file pone.0183563.s001.pdf]

Fig. S1

A

WT PGM3

```
MDLGAITKYSALHAKPNGLILQYGTAGFRTKAEHLDHVMFRMGLLAVLRSKQTKSTIGVM
VTASHNPEEDNGVKLVDPLGEMLAPSWEEHATCLANAEEQDMQRVLIDISEKEAVNLQQD
AFVVIGRDTRPSSEKLSQSVIDGVTVLGGQFHDYGLLTTPQLHYMVYCRNTGGRYGKATI
EGYYQKLSKAFVELTKQASCSGDEYRSLKVDCANGIGALKLREMEHYFSQGLSVQLFNDG
SKGKLNHLCGADFVKSHQKPPQGMEIKSNERCCSFDGDADRIVYYYHDADGHFHLIDGDK
IATLISSFLKELLVEIGESLNIGVVQTAYANGSSTRYLEEVMKVPVYCTKTGVKHLHHKA
QEFDIGVYFEANGHGTALFSTAVEMKIKQSAEQLEDKKRKAAKMLENIIDLFNQAAGDAI
SDMLVIEAILALKGLTVQQWDALYTDLPNRQLKVQVADRRVISTTDAERQAVTPPGLQEA
INDLVKKYKLSRAFVRPSGTEDVVRVYAEADSQESADHLAHEVSLAVFQLAGGIGERPQP
GF
```

Rearranged PGM3 (Grouped Segments)

```
MDLGAITKYSALHAKPNGLILQYGTAGFRTKAEHLDHVMFRMGLLAVLRSKQTVNLQQDA
FVVIGRDTRPSSEKLSQSVIDGVTVLGGQFHDYGLLTTPQLHYMVYCRKSTIGVMVTASH
NPEEDNGVKLVDPLGEMLAPSWEEHATCLANAEEQDMQRVLIDISEKEANTGGRYGKATI
EGYYQKLSKAFVELTKQASCSGDEYRSLKVDCANGIGALKLREMEHYFSQGLSVQLFNDG
SKGKLNHLCGADFVKSHQKPPQGMEIKSNERCCSFDGDADRIVYYYHDADGHFHLIDGDK
IATLISSFLKELLVEIGESLNIGVVQTAYANGSSTRYLEEVMKVPVYCTKTGVKHLHHKA
QEFDIGVYFEANGHGTALFSTAVEMKIKQSAEQLEDKKRKAAKMLENIIDLFNQAAGDAI
SDMLVIEAILALKGLTVQQWDALYTDLPNRQLKVQVADRRVISTTDAERQAVTPPGLQEA
INDLVKKYKLSRAFVRPSGTEDVVRVYAEADSQESADHLAHEVSLAVFQLAGGIGERPQP
GF
```

B

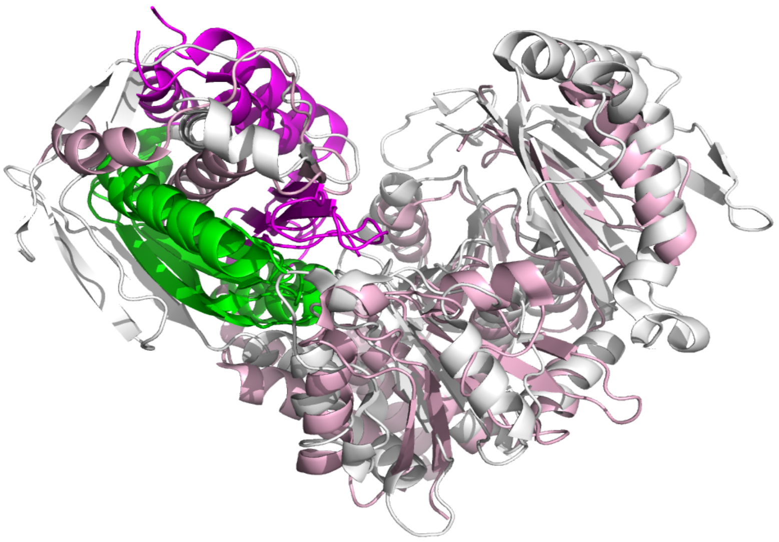

Fig. S1. Details of the sequence modification made to human PGM3.
(A) Sequences of wild-type and human PGM3 and the modified version used for sequence alignments, showing the transposed regions (see text). Residues highlighted in magenta were swapped with those in green. **(B)** Superposition of human PGM1 (white) and the PGM3 homolog from C. albicans (PDB ID 2DKA) (pink). Green/magenta highlight the regions affected by circular permutation (and swapped in the amino acid sequence), showing how they are structurally conserved despite the different sequence connectivity in PGM3.
