# Supplementary material for: Sequence-structure relationships, expression profiles, and disease-associated mutations in the paralogs of phosphoglucomutase 1
Source: PLoS One. 2017 Aug 24;12(8):e0183563. doi: 10.1371/journal.pone.0183563 (PMC5570346; doi:10.1371/journal.pone.0183563)
Supplement: S2 Fig — Data are binned into low (light blue), medium (orange), or high (red) expression. Dark blue indicates no measureable expression; tissues with no expression for any paralog were omitted. (PDF) [file pone.0183563.s002.pdf]

Fig. S2

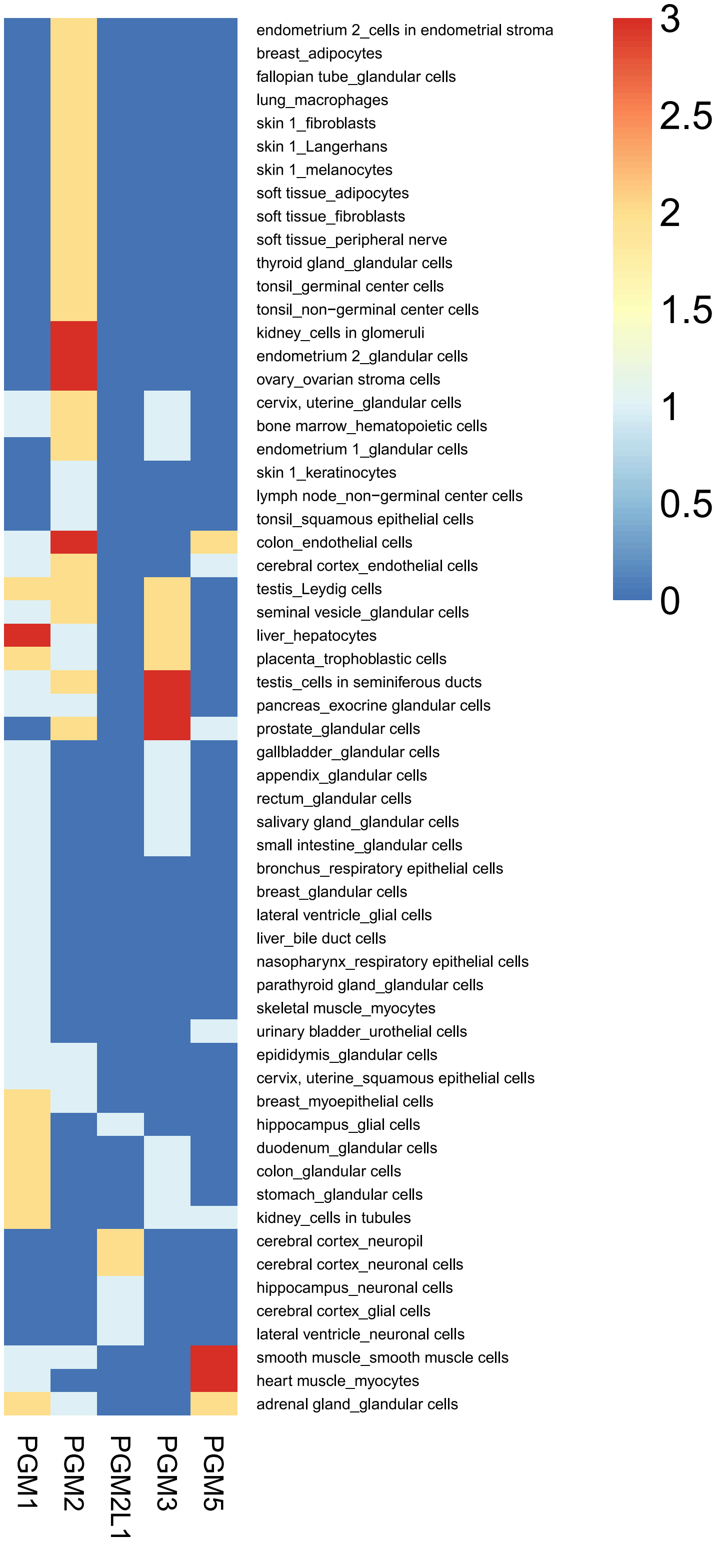

Fig. S2. Protein expression data for the PGM paralogs from the Human Protein Atlas (ref) showing data on a wide range of tissues. Data are binned into low (light blue), medium (orange), or high (red) expression. Dark blue indicates no measureable expression; tissues without expression for any paralog were omitted.
