# Supplementary material for: Sequence-structure relationships, expression profiles, and disease-associated mutations in the paralogs of phosphoglucomutase 1
Source: PLoS One. 2017 Aug 24;12(8):e0183563. doi: 10.1371/journal.pone.0183563 (PMC5570346; doi:10.1371/journal.pone.0183563)
Supplement: S1 Table — (PDF) [file pone.0183563.s005.pdf]

S1 Table. No. of expected vs. observed variants of the PGM1 paralogs and their evolutionary constraint scores

	PGM1	PGM2	PGM2L1	PGM3	PGM5
**Expected no. variants**	208.2	175.6	191.6	155.6	171.7
**Observed no. variants**	206	207	128	150	139
**Constraint scores**	0.08	-1.16	2.25	0.22	1.22

Variant numbers and constraint scores obtained from the ExAc database (see text).

Genes with constraints scores > 0.9 are considered highly intolerant to substitution.
